# Conservation Priorities and Demographic History of *Saussurea involucrata* in the Tianshan Mountains and Altai Mountains

**DOI:** 10.3390/life13112209

**Published:** 2023-11-14

**Authors:** Lin Hu, Ting Lu, Xiyong Wang, Jiancheng Wang, Wei Shi

**Affiliations:** 1 State Key Laboratory of Desert and Oasis Ecology, Key Laboratory of Ecological Safety and Sustainable Development in Arid Lands, Xinjiang Institute of Ecology and Geography, Chinese Academy of Sciences, Urumqi 830011, China; hulin54@163.com (L.H.); wangxy@ms.xjb.ac.cn (X.W.); 2 College of Forestry and Landscape Architecture, Xinjiang Agricultural University, Urumqi 830011, China; luting0909@126.com; 3 Turpan Eremophytes Botanic Garden, The Chinese Academy of Sciences, Turpan 838008, China

**Keywords:** *Saussurea involucrata*, Tianshan Mountains and Altai Mountains, chloroplast and nuclear regions, conservation

## Abstract

Rare and vulnerable endemic plants represent different evolutionary units that occur at different times, and protecting these species is a key issue in biological protection. Understanding the impact of the history of endangered plant populations on their genetic diversity helps to reveal evolutionary history and is crucial for guiding conservation efforts. *Saussurea involucrata*, a perennial alpine species mainly distributed in the Tianshan Mountains, is famous for its medicinal value but has become endangered due to over-exploitation. In the present study, we employed both nuclear and chloroplast DNA sequences to investigate the genetic distribution pattern and evolutionary history of *S. involucrata*. A total of 270 individuals covering nine *S. involucrata* populations were sampled for the amplification and sequencing of nrDNA Internal Transcribed Spacer (ITS) and chloroplast *trn*L-*trn*F, *mat*K and *ndh*F-*rpl*32 sequences. Via calculation, we identified 7 nuclear and 12 plastid haplotypes. Among the nine populations, GL and BA were characterized by high haplotype diversity, whereas BG revealed the lowest haplotype diversity. Molecular dating estimations suggest that divergence among *S. involucrata* populations occurred around 0.75 Ma, coinciding with the uplift of Tianshan Mountains. Our results reveal that both isolation-by-distance (IBD) and isolation-by-resistance (IBR) have promoted genetic differentiation among populations of *S. involucrata*. The results from the ecological niche modeling analyses show a more suitable habitat for *S. involucrata* in the past than at present, indicating a historical distribution contraction of the species. This study provides new insight into understanding the genetic differentiation of *S. involucrata*, as well as the theoretical basis for conserving this species.

## 1. Introduction

The Tianshan Mountains and the Altai Mountains, located in the arid periphery of the Junggar Basin in northwestern China, are part of the Central Asian and High Asian Mountain systems [1,2]. The Qinghai–Tibet Plateau is the highest plateau in the world. It has undergone complex geological events and environmental fluctuations, especially the rapid uplift since the late Pliocene and subsequent Quaternary climate oscillations. Climate change in the history of the Tibetan Plateau has also affected the structure and evolution of its flora and fauna [3,4,5,6]. It can be reflected in the spatial diversity based on the genetic structure and evolutionary process of plants, along with Qinghai–Tibet Plateau uplift and climate change, especially in studying the geographical distribution pattern and genetic structure of endemic plants in the Qinghai–Tibet Plateau, allowing us to explore the effects of Qinghai–Tibet Plateau uplift and climate change on the spatial genetic structure and evolution of plants [7,8]. As a part of the flora of the Qinghai–Tibet Plateau, this area is rich in endemic species, but there are relatively few studies on species in this area [9]. There are four stages of the initial uplift of the Tianshan Mountains and the subsequent major uplifts along with the uplift of the Qinghai–Tibet Plateau, which affected the genetic diversity of plants in the regions significantly. The uplift of these mountains is the result of the Cenozoic India–Asia collision and the rise of the Qinghai–Tibet Plateau [10,11]. Plant genetic diversity is the ability to maintain adaptability and respond to environmental changes. It is the main concern of conservation genetics. To protect endangered species, the main geographical lineages should be protected [12]. It is very important to study the conservation genetics of endangered plants for the formulation of protection strategies and protection management [13]. The protection of rare and endangered species should focus on endemic species with limited geographical distribution [14]. Through the study of the conservation genetics of endangered plants endemic to the Qinghai–Tibet Plateau, effective protection strategies can be proposed [15,16]. Based on the systematic geographical study of endangered plants endemic to arid northwestern China, we can better understand the evolution history of species in this area and put forward protection strategies [8,17]. The Tianshan Mountains and the Altai Mountains are important parts of the arid region in northwestern China. However, there are few studies on the conservation genetics of endangered alpine endemic species in this region. *Saussurea involucrata* is only distributed in the Tianshan Mountains and Altai Mountains in China. The study of conservation genetics can provide a reference for its protection research.

*Saussurea involucrata* is an endemic alpine plant, just distributed in the Tianshan Mountains and Altai Mountains with an altitude of about 2400–4100 m. It has been listed as a national secondary protected plant [18,19] (http://www.Iplant.cn/bhzw/info/1102 (accessed on 7 September 2021)). As a traditional medicinal plant, *S. involucrata* has been used for dispelling wind to eliminate dampness, eliminating inflammation, promoting blood circulation and relieving pain [20,21]. In recent years, the pharmacological activity of *S. involucrata* in anti-cancer has been gradually discovered and deeply explored by researchers [22,23]. *Saussurea involucrata* has suffered from excessive excavation and habitat destruction for a long time and has not been effectively and reasonably protected, resulting in a sharp decline in its resources [24]. In order to avoid resource depletion or even extinction, it is urgent to formulate appropriate protection strategies to reduce resource plunder and habitat destruction caused by human activities. By analyzing the genetic diversity and population differentiation of five populations of *S. involucrata* distributed in the western Tianshan Mountains, we should protect them in situ and give priority to the protection of *S. involucrata* in the Bayinbuluke area [25]. 

In order to further analyze the distribution pattern and population history of *S. involucrata*, in this study, we increased the sampling points of *S. involucrata* to cover the Tianshan Mountains and Altai Mountains. We comprehensively analyzed the population structure, population history, ancestral population reconstruction, isolation-by-distance (IBD) and isolation-by-resistance (IBR). Our analyses reveal the differentiation process of *S. involucrata* across and within geographic regions. This study provides new insights into the differentiation of *S. involucrata* and the theoretical basis for the establishment of effective protection strategies for this species. 

## 2. Materials and Methods

### 2.1. Population Collection and DNA Data Generation

Nine populations of *S. involucrata* were sampled. A total of 270 samples were collected from each population in the Tianshan Mountains and Altai Mountains. The longitude, latitude and altitude were recorded during collection. Fresh leaves were collected, immediately dried on silica gel and stored at room temperature for DNA extraction. DNA was extracted from silica gel dried leaves via the CTAB method [26].

Initially, ten plastid DNA segments (*trn*S-*trn*G, *atp*B-*rbc*L, *trn*Q-*rps*16, *rpl*32-*trn*L, *psb*K-*psb*I, *psb*A-*trn*H, *rps*16, *rps*12-*rp*l20 and *ycf*6-*psb*M) were screened for genetic variations [25,27], but only *trn*L-*trn*F, *mat*K and *ndh*F-*rpl*32 were found to have effective levels of variation. For the amplification of nuclear regions, we selected the gene ITS1–4. ITS1–4 was successfully amplified and exhibited sufficient variation in preliminary experiments. PCR amplification and sequencing of both strands were performed using previously published methods for these regions. PCR amplification and sequencing of the ITS region were implemented with ITS1-4 primers. All PCR products were stained with SYBR on 1.5% agarose gel electrophoresis to check the amplification. PCR amplification and sequencing of *cp*DNA were performed using three primers: *trn*L-*trn*F, *mat*K and *ndh*F-*rpl*32. In addition to the annealing temperature of 55 ℃ and the total volume of 50 μL, the PCR protocol was the same as the ITS amplification protocol.

### 2.2. Population Structure and Diversity

DNA sequences were aligned using ClustalX and manually checked in Bioedit. DnaSP v 5 was used to calculate haplotypes, haplotype diversity (Hd) and nucleotide diversity (π) [28]. Haplotypes defined by DnaSP were used to construct a media-joining network under default parameters in NETWORK. ArcMap 9.3 software in the ArcGIS v 9.3 software package, combined with sample collection information, was used to draw a haplotype map.

**Figure 1:**
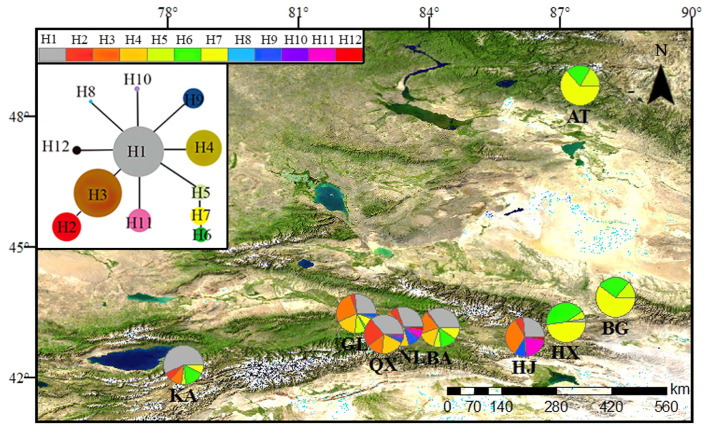
Geographic distributions and frequencies of the *cp*DNA gene regions detected in the *S. involucrata* populations in the Tianshan Mountains and Altai Mountains, with their haplotype networks (H1–H12) constructed by using TCS 1.21.

**Figure 2:**
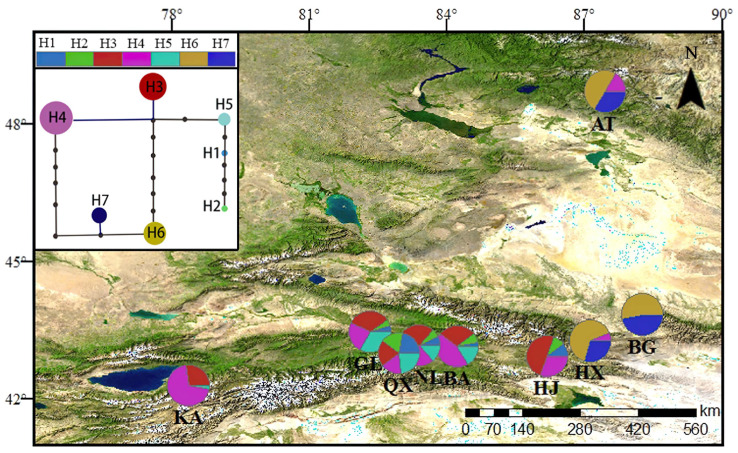
Geographic distributions and frequencies of the nrDNA gene regions detected in the *S. involucrata* populations in the Tianshan Mountains and Altai Mountains, with their haplotype networks (H1–H7) constructed by using TCS 1.21.

A haplotype network and unrooted and statistical parsimony figure were drawn using TCS version 1.21. These analyses showed the genealogical relationships of haplotypes for *cp*DNA (Figure 1) and nrDNA (Figure 2).

### 2.3. Divergence Time of Haplotypes

To estimate the divergence times of phylogenetic lineages, the differentiation of time estimation for *cp*DNA haplotypes and nrDNA haplotypes was performed. According to Emkeetal’s research settings, the base exchange rate of *trn*L-*trn*F, *mat*K and *ndh*F-*rpl*32 was 1.45 × 10^−9^ s/s/y, and the base exchange rate of ITS was 6.1 × 10^−9^ s/s/y. Using Tracer1.7.2 to check the output log file to check whether the ESS is greater than 200. Using TreeAnnotator 1.5.4 to burn the top 20 % unstable tree final file [29].

Bayesian skyline analysis was performed in the BEAST 1.7.0 version to show the population status of the population and estimate past population dynamics [30]. the BSP was analyzed and plotted in the R language.

### 2.4. Isolation-by-Distance (IBD) and Isolation-by-Resistance (IBR) Analyses

In order to study the role of geography and environment in shaping spatial genetic differentiation, we tested spatial isolation (IBD) and isolation resistance (IBR). The normalized pairwise genetic distance FST/(1-FST) between populations was calculated using ‘hierfstat’. The geographical distance was calculated by using the R package ‘land boundary’ [31] according to the latitude and longitude information of the population. Using topo Dist in the R package topo Dist, the resistance distance was calculated based on the elevation grid layer and the latitude and longitude information of the site. The number of moving directions between units was set to 8 [32].

### 2.5. Population Historical Developments

DnaSP v 5 was used to construct the mismatch distribution curve of *S. involucrata* [33] to test historical demographic expansion. If a unimodal curve appeared in the mismatch distribution analysis, it was considered that the overall had recently expanded; if the curve was bimodal or multimodal, it was considered that the population had not expanded recently but was in a dynamic equilibrium state [34].

## 3. Results

### 3.1. Population Structure and Diversity

**Figure 3:**
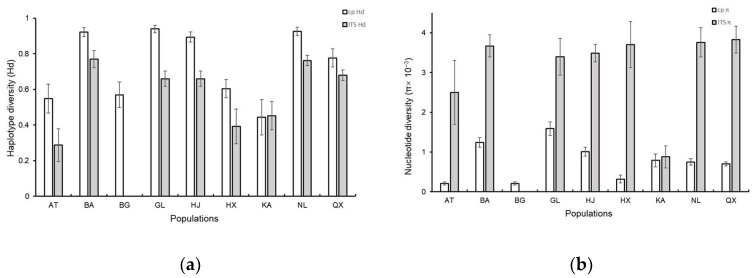
(**a**) *Cp* DNA and ITS haplotype diversity (Hd) of 9 populations of *S. involucrata*; (**b**) C*p* DNA and ITS nucleotide diversity (π) of 9 populations of *S. involucrata*.

A total of 12 chloroplast haplotypes (Figure 1) and 7 nuclear haplotypes (Figure 2) were detected in nine *S. involucrata* populations. The chloroplast haplotype H1 was the most widely distributed haplotype, which was found in seven *S. involucrata* populations (Figure 1). Each haplotype of chloroplast genes is related to at least another haplotype, and haplotype H1 revealed the highest number of connections. Nuclear haplotypes H4 and H3 are widely distributed haplotypes, where haplotype H4 is distributed in eight populations, and H3 is distributed in six populations. Among the seven haplotypes probed by the nuclear ITS1-4 gene, two haplotypes, H6 and H7, are rare haplotypes, and these two haplotypes are only distributed in three populations: Altai (AT), Bogda (BG) and Haxionggou (HX) (Figure 2). Each of the nuclear haplotypes is related to the other haplotypes. *Saussurea involucrata* showed high levels of haplotype (Figure 3a) and nucleotide diversity (Figure 3b). According to the *cp*DNA data of *S. involucrata*, the Narat (NL) population had the highest Hd value (Figure 3a), and the Sclerophore (GL) population had the highest nucleotide diversity (π) value (Figure 3b). Based on the analyses of the nuclear ITS1-4 gene, the Bayinbuluke (BA) population had the highest Hd values (Figure 3a), and the Chasi (Q X) population had the highest nucleotide diversity (π) values (Figure 3b). The total haplotype diversity (Hd) of *cp*DNA was 0.895, ranging from 0.886 to 0.904, and the total nucleotide diversity (π) of *cp*DNA was 1.26, ranging from 1.24 to 1.28. The haplotype diversity (Hd) in the ITS1-4 region was 0.813, ranging from 0.804 to 0.822, and π was 6.13, ranging from 5.98–6.28, which was much higher than that of the chloroplast genes.

### 3.2. Genetic Divergence History

**Figure 4:**
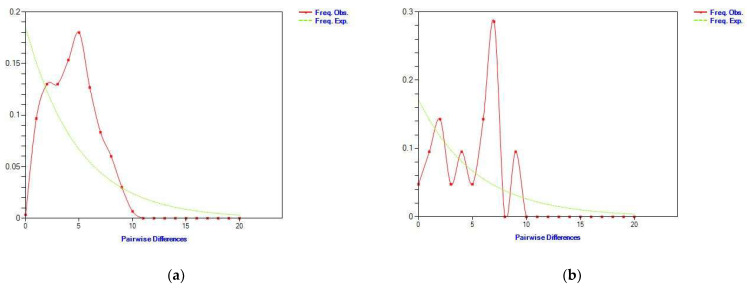
(**a**) *Cp*DNA mismatch distribution curve of *S. involucrata*; (**b**) ITS mismatch distribution curve of *S. involucrata*. The solid line and the dashed line represent observed values and expected values, respectively.

**Figure 5:**
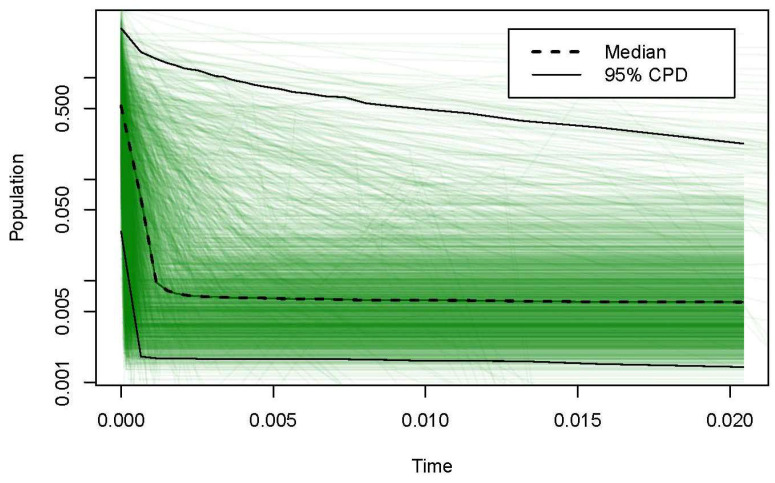
Neutrality test of *S. involucrata.* The 95% confidence interval and the median value are represented by the real line and the imaginary line, respectively.

Based on the analyses of chloroplast gene mismatch distribution (Figure 4a) and nuclear gene mismatch distribution (Figure 4b), the results show that the mismatch distribution curve was the multi-peak type, and the expected value was inconsistent with the observed values and the species expansion model, indicating that there was no significant population expansion or bottleneck effect. In recent years, *S. involucrata* has not experienced rapid expansion. The neutral test results show that the population of *S. involucrata* had a history of a stable population size and had not experienced population expansion and a continuous growth pattern (Figure 5).

### 3.3. Isolation-by-Distance (IBD) and Isolation-by-Resistance (IBR) Analyses

**Figure 6:**
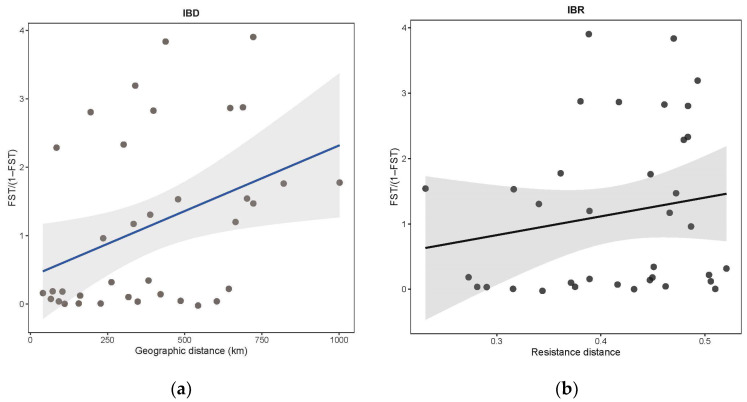
Isolation by geography and environment in *S. involucrata*. (**a**) Isolation-by-distance (IBD); (**b**) Isolation-by-resistance (IBR). Gray shading around each regression line represents the 95% confidence interval.

By quantifying the contribution of IBD (Figure 6a) and IBR (Figure 6b) to the spatial and genetic distribution of *S. involucrata*, the genetic differentiation pattern among *S. involucrata* populations was explained. The Mantel test results show that the genetic structure of the population could be explained by IBD. Compared with IBR, IBD can explain the spatial genetic differentiation of *S. involucrata* more effectively. This result is also quite consistent with the distribution space of *S. involucrata* in reality. The existing *S. involucrata* populations are limited to fragmented habitats at a certain altitude, and the geographical distance between populations is large. This distribution pattern can easily cause a sharp decrease in gene flow between groups, thereby increasing the genetic distance between groups.

## 4. Discussion

### 4.1. High Genetic Diversity 

Based on the *cp*DNA intergenic spacers (*trn*L-F, *mat*K and *ndh*F-*rpl*32) and nrDNA (ITS1-4) of *S. involucrata*, our results show that the nine natural populations of *S. involucrata* had high haplotype diversity (Figure 3a) and nucleotide diversity (Figure 3b) at the species level, but there were some differences in haplotype diversity and nucleotide diversity among different populations. Habitat fragmentation usually leads to a decrease in genetic diversity among populations and a huge difference in genetic diversity among populations [35]. The difference in genetic diversity among different populations of *S. involucrata* may be related to the habitat fragmentation of *S. involucrata* as a typical alpine plant. High genetic diversity may be related to its reproductive system, biological characteristics, seed dispersal ability, geographical distribution and population size [36]. In general, widely distributed species that are perennial with a high outcrossing rate and diverse seed dispersal methods have a high level of genetic diversity. *Saussurea involucrata* is a perennial plant, and its florets have male pre-maturation and herkogamy. This spatial structure is conducive to cross-pollination [37]. *Saussurea involucrata* has a variety of diffusion methods based on wind media, water media and animal transmission. Its seeds have long crown hairs that can be spread by wind and can be spread far away. The biological characteristics of *S. involucrata* reproductive systems can also explain the high genetic diversity of *S. involucrata* natural populations adapting to extreme alpine environments. The genetic diversity of *S. involucrata* is higher than that of other alpine plants occurring in this area and adjacent areas, such as *Saussurea obvallata* (Ht = 0.454, Hs = 0.275) [38] and *Saussurea medusa* (He = 0.2757) [39]. Different physiological characteristics and habitats of the species may cause these results.

### 4.2. The Conserved Center of S. involucrata 

The refuge is less affected by climate fluctuations and can maintain rich genetic diversity among populations. Sites with high levels of genetic variation and special haplotypes may be refuges for this species [40]. The genetic diversity of the wild apricot population in the Tianshan area remained at a high level (He = 0.6109, I = 1.2208), which confirmed that the Tianshan wild apricot was the origin center of the cultivated apricot [41]. In this study, nine populations of *S. involucrata* were collected from the Tianshan Mountains and Altai Mountains. The genetic diversity of *S. involucrata* in the western Tianshan Mountains was higher and had special haplotypes. Specifically, the *S. involucrata* population in the Bayinbuluke area in the western Tianshan Mountains had higher genetic diversity and had special haplotypes (i.e., H5–H8) (Figure 1). Therefore, we speculate that the Bayinbuluke area may be the genetic differentiation center of *S. involucrata*. Notably, the area is also identified as a refuge for Iris tectorum [42]. Based on the founder effect, the genetic diversity of the original population was higher than that of the population formed by migration and diffusion, and there were more unique haplotypes [43]. Due to water vapor from westerly airflow into the valley, the species in the northwest arid area are less affected by the barrier of the Qinghai–Tibet Plateau and its surrounding mountains, and the western Tianshan Mountains and Altai Mountains are more humid than the east [9]. In this study, the results of chloroplast genes and ribosomal genes show that the genetic diversity of *S. involucrata* in the western Tianshan Mountains was higher (Figure 3) and had special haplotypes (Figure 1 and Figure 2). Therefore, this area may be the origin center of *S. involucrata*.

Our data show that the *cp*DNA and nrDNA mismatch distribution curves of *S. involucrata* were multi-peak (Figure 4), and the neutral test results show that *S. involucrata* conformed to the neutral evolution model (Figure 5), suggesting that the *S. involucrata* population had a history of a relatively stable population size. The mismatch distribution curve and neutral test of *S. involucrata* showed that the population size of *S. involucrata* was relatively stable and did not expand in the near future, which was similar to the results of the study in the western Tianshan Mountains of *S. involucrata* [25]. This may be related to the high level of genetic diversity, the fragmentation of habitat range, or the distance isolation of *S. involucrata*, thus maintaining the balance of the population. There is a general lack of phytogeographical studies in the Tianshan and Altai Mountains [9], but the region’s flora is mainly affected by Pleistocene glacial-dry glacial to interglacial-wet period changes [44]. In this study, the nuclear haplotypes from the nine populations of *S. involucrata* from the Tianshan and Altai Mountains showed two independent lineages. The H6 and H7 haplotypes were only distributed in the Altai and eastern Tianshan Mountains (HX, BG and AT), and another lineage was mainly distributed in the western Tianshan Mountains (Figure 2). The population structure of *S. involucrata* is similar to that of *Clematis sibirica* [45] and has independent lineages in the Altai and western Tianshan Mountains. Based on the rooted phylogenetic network, the nrDNA haplotype of *S. involucrata* was missing during the evolutionary process (Figure 2).

**Figure 7:**
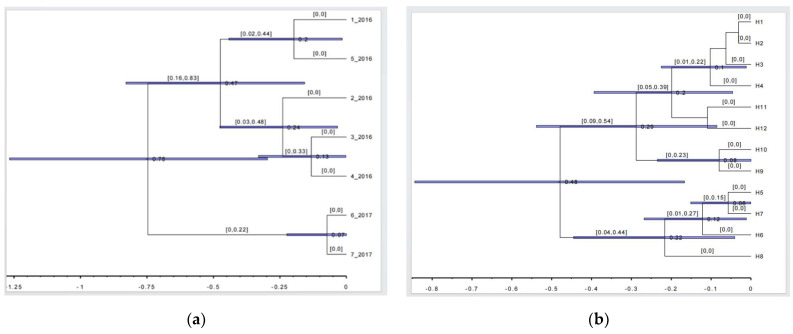
(**a**) The geographical lineage of nrDNA haplotypes of *S. involucrata* was divided and dated based on aggregation analysis; (**b**) The geographical lineage of *cp*DNA haplotypes of *S. involucrata* was divided and dated based on aggregation analysis.

The Cenozoic intracontinental orogeny in the Tianshan Mountains and Central Asia was affected by the Himalayan collision and the uplift of the Qinghai–Tibet Plateau [46]. The rapid uplift of the Tianshan Mountains not only changed the topography but also led to the intensification of drought in the surrounding areas [47]. Geology and climate change have influenced the genetic structure differentiation of plants in this region [48,49]. The populations of *S. involucrata* in the eastern Tianshan Mountains and Altai Mountains have special nuclear gene haplotypes H6 and H7 (Figure 2), which were differentiated from the *S. involucrata* population distributed in the western Tianshan Mountains during the Pleistocene period (0.75 Ma) (Figure 7a). The recent uplift time of the Tianshan Mountains can be traced back to the Pleistocene (0.73 Ma) [11]. We estimate that the time of the haploid differentiation of the *S. involucrata* nuclear gene was consistent with the time of the Tianshan uplift. It shows that, after the early spread of *S. involucrata* to the Altai Mountains and eastern Tianshan Mountains, the uplift of the Tianshan Mountains changed the terrain, and geographical isolation promoted the early differentiation of nuclear gene haplotypes H6 and H7. 

The arrival of glaciers during the Pleistocene (0.01–2.59 Ma) made the geological age officially enter the cold Quaternary. Large-scale iceberg movements occurred in the high, middle and low latitudes of the Northern Hemisphere. The alternation of glacial and interglacial cycles accelerated the intraspecific differentiation of plants [43,50,51,52]. The largest glaciation in the Tibetan Plateau and adjacent areas began at about 1.2 Ma and reached a maximum between 0.8 and 0.6 Ma [53,54]. The nuclear gene haplotype differentiation (0.47 Ma) and chloroplast haplotype differentiation (0.48 Ma) of *S. involucrata* began in the Middle Pleistocene. The estimated differentiation time of its lineage is later than the maximum glacial period. The extremely low temperature during the glacial period may have caused obstacles to gene flow between geographically isolated *S. involucrata* populations, and gene flow was cut off or restricted. It promoted the generation of regional special haplotypes [55]. We believe that the intraspecific haplotypes of *S. involucrata* rapidly diversified, and the divergence time was roughly the same as that of many plants, such as *Cyananthus delavayi* (0.49 Ma) and *Pomatosace filicula* (0.53 Ma), affected by the Qinghai–Tibet Plateau glacial period [52,56]. In the Pleistocene, the Tianshan Mountains experienced three different glacial and interglacial cycles caused by climate turmoil, which played an important role in the distribution and genetic differentiation of species in this area [57,58,59], which also profoundly affected the genetic structure differentiation of *S. involucrata*. Many studies on the phylogenetic structure differentiation of alpine plant populations have proved that the uplift of the Qinghai–Tibet Plateau and Quaternary glacial oscillation have had a great impact on the distribution and evolution history of plant species [60,61]. We speculate that *S. involucrata* survived in the eastern Tianshan Mountains, Altai Mountains and western Tianshan Mountains when the Quaternary glacial climate repeatedly oscillated. At the same time, the uplift of Tianshan Mountains seriously hindered gene exchange between populations, resulting in the fragmentation of the distribution of *S. involucrata* in the current habitat.

### 4.3. Protection Recommendations 

In order to develop targeted conservation management strategies, it is necessary to understand the genetic structure of threatened species [62]. Maintaining genetic diversity is a key issue in protecting and managing the long-term survival of endangered species [12]. With global warming, the Tianshan snowline rises, and the growth area of *S. involucrata* also shrinks sharply. Due to excessive excavation and the destruction of the natural environment, the distribution area and population size of *S. involucrata* have decreased sharply, resulting in the loss of the germplasm resources of *S. involucrata*. In view of the fact that *S. involucrata* is a perennial one-time flowering and fruiting plant, it is difficult to restore its genetic diversity after it is disturbed by human activities. The protection of *S. involucrata* germplasm resources are important. Habitat reduction and human destruction will cause population fluctuations, and ultimately showing a low level of diversity, high population differentiation and a high level of inbreeding in genetics. Inbreeding in small-scale and fragmented populations may lead to the loss of genetic variation, which seriously affects the adaptability of threatened species to environmental changes [63]. Therefore, maintaining an effective population size and genetic diversity of *S. involucrata* should be a priority in the conservation plan. This study increases our understanding of the possible main factors affecting the genetic composition of the *S. involucrata* population, thus benefiting its population protection. The *cp*DNA haplotypes of *S. involucrata* were closely related, but some haplotypes were missing, indicating that there may be a loss of genetic diversity. Due to geographical isolation, each *S. involucrata* population should be protected as an independent unit. Among the nine populations of *S. involucrata* in the Tianshan and Altai Mountains, the genetic diversity of *S. involucrata* in the Bayinbuluke area is high, and there are many special haplotypes that should be given priority protection. Based on in situ conservation, seed preservation can also be carried out, and a few individuals in the unique haplotype population and plants with special traits can be collected and transferred to the resource nursery for protection. The natural growth cycle of *S. involucrata* is 5–6 years. The devastating root digging and flowering of the snow lotus has seriously affected its natural reproduction. We need a comprehensive artificial propagation strategy for *S. involucrata*, including seed germination, large-scale seedling production, grafting and tissue culture (i.e., somatic embryogenesis) programs, to avoid collecting plants from natural populations. Finally, all the protection work is inseparable from the support of the residents‘ community, and it is also necessary to enhance residents’ awareness of natural protection.

## 5. Conclusions

In this study, the effective protection of *S. involucrata* is proposed by exploring the factors affecting the population history and genetic structure of *S. involucrata*. The strong uplift of the Tianshan Mountains in the Pleistocene and the alternation of the Quaternary glacial and interglacial periods played an important role in the genetic differentiation of *S. involucrata* populations. The results of the niche model analysis show that geographical isolation could promote the genetic differentiation of *S. involucrata* compared with IBR. The geographical distance between populations of *S. involucrata* is far, which can easily lead to a sharp decrease in gene flow between populations, thus increasing the genetic distance between populations. The *S. involucrata* distributed in the Tianshan Mountains and Altai Mountains is affected by the uplift of the Tianshan Mountains, resulting in the fragmentation of its distribution in the current habitat. *Saussurea involucrata* is divided into two lineages, the Tianshan Mountains and Altai Mountains, which seriously hinders gene exchange between populations. The study of the gene differentiation and genetic structure of *S. involucrata* provides evidence for future research on the response of alpine endemic plants to Quaternary geological and climatic events in the Tianshan and Altai regions. The population of *S. involucrata* distributed in the western Tianshan Mountains has high genetic diversity. In order to avoid the loss of endangered species of *S. involucrata* germplasm resources, priority should be given to the protection of *S. involucrata* in this area. In order to further protect *S. involucrata* effectively, *S. involucrata* in the Bayinbuluke area deserves further study.

## Data Availability

The data presented in this study are available on request from the corresponding author.
